# Large-Scale Structure of a Network of Co-Occurring MeSH Terms: Statistical Analysis of Macroscopic Properties

**DOI:** 10.1371/journal.pone.0102188

**Published:** 2014-07-09

**Authors:** Andrej Kastrin, Thomas C. Rindflesch, Dimitar Hristovski

**Affiliations:** 1 Faculty of Information Studies, Novo mesto, Slovenia; 2 Lister Hill National Center for Biomedical Communications, National Library of Medicine, Bethesda, Maryland, United States of America; 3 Institute for Biostatistics and Medical Informatics, Faculty of Medicine, University of Ljubljana, Ljubljana, Slovenia; Universidad Veracruzana, Mexico

## Abstract

Concept associations can be represented by a network that consists of a set of nodes representing concepts and a set of edges representing their relationships. Complex networks exhibit some common topological features including small diameter, high degree of clustering, power-law degree distribution, and modularity. We investigated the topological properties of a network constructed from co-occurrences between MeSH descriptors in the MEDLINE database. We conducted the analysis on two networks, one constructed from all MeSH descriptors and another using only major descriptors. Network reduction was performed using the Pearson's chi-square test for independence. To characterize topological properties of the network we adopted some specific measures, including diameter, average path length, clustering coefficient, and degree distribution. For the full MeSH network the average path length was 1.95 with a diameter of three edges and clustering coefficient of 0.26. The Kolmogorov-Smirnov test rejects the power law as a plausible model for degree distribution. For the major MeSH network the average path length was 2.63 edges with a diameter of seven edges and clustering coefficient of 0.15. The Kolmogorov-Smirnov test failed to reject the power law as a plausible model. The power-law exponent was 5.07. In both networks it was evident that nodes with a lower degree exhibit higher clustering than those with a higher degree. After simulated attack, where we removed 10% of nodes with the highest degrees, the giant component of each of the two networks contains about 90% of all nodes. Because of small average path length and high degree of clustering the MeSH network is small-world. A power-law distribution is not a plausible model for the degree distribution. The network is highly modular, highly resistant to targeted and random attack and with minimal dissortativity.

## Introduction

The proliferation of scientific knowledge during the past decades makes it difficult even for domain experts to keep abreast of the relevant information in their specific field of interest. Life sciences literature, nowadays referred to as the bibliome, is highly massive and of unprecedented volume and complexity. At the time of this writing, the MEDLINE database [1] contains over 23 million bibliographic citations with a continuous growth rate of about 2,000–4,000 citations per day.

Associations between entities based on co-occurrence of biomedical terms, such as chemical substances, biological processes, diseases or genes constitute an important part of knowledge representation. A co-occurrence approach is built on the assumption that biomedical concepts occurring together in the same title or abstract are in some way biologically related [2], [3]. Simple linkage between concepts can be further extended by the number of times a concept is found in a document or by closeness between one concept and another concept in a sentence [4]. Literature mining technologies complement information extracted from structured biomedical sources (e.g., GeneOntology) by providing researchers with more relevant and interpretable knowledge. A plethora of applications have been developed exploiting co-occurrence for mining interesting patterns in biomedical resources (e.g., BITOLA [5], iHOP [6], AliBaba [7], EBIMed [8], FACTA [9], PLAN2L [10], STRING [11], LAITOR [12]).

Analyzing big relational datasets requires innovative methodological approaches beyond the basic exploratory mining. Recent years have seen an increasing interest in the study of large, real-world, complex networks, in which graph theory is used to model the relationships between the entities [13]. Knowledge of a domain can be viewed as a set of concepts along with the relations between them [14]. Interactions between concepts can be described in terms of a graph, consisting of nodes and edges, where the former represent concepts and the latter represent their relationships. The graph thus created can be used to decipher the structure of a complex network and help to identify statistically relevant properties and interesting patterns. Empirical evidence has revealed that complex networks exist on many scales and spanning different fields of reality, including statistical mechanics [15], cognitive science [16], and cell biology [17]. Real-world networks show various nontrivial topological properties that do not occur in simple or random networks [18], [19]. The key to understanding such complex systems are the mechanisms that determine the topology of their induced networks.

Complex real-world networks are characterized by two major distinguishing properties: strong local clustering and short global distances between nodes [18]. High local clustering refers to dense local clusters of connections yet sparse interconnections between clusters. Granovetter [20], [21] showed in his seminal paper that society is fragmented into clusters of individuals having similar characteristics, and clustering is a general feature of many other types of networks (e.g., blogosphere, online social networks). The high degree of clustering indicates that if nodes X and Y are linked to node Z, then X and Y are also likely to be linked to each other. Average clustering over the set of nodes in a real-world network is significantly higher in comparison to a random network with the same number of nodes and edges. It has also been demonstrated that networks with high clustering have a hierarchical organization and modular structure [22]. In addition to strong local clustering, a real-world network is characterized by small average shortest path length, making it possible to connect any pair of nodes by traversing only a few connections. This means that all nodes of a large network are connected through relatively few intermediate steps, despite the fact that most nodes maintain only a limited number of connections, mostly within a clique of neighbors.

A network with these two properties is called a small-world network. The idea of small-world networks initially emerged through a famous experiment performed by Milgram [23] in the late 1960s. Milgram showed that the average number of acquaintances separating any two people in the USA is about six. This observation was later popularized as the ‘six degrees of separation’ phenomenon [24]. It has been demonstrated that average shortest path length between node pairs in a network grows logarithmically with network size [18]. Studies have shown that the Web, scientific collaboration of research papers, film actors, and general social networks are all examples of networks with small-world properties.

In addition to being characterized as small-world networks, real-world networks have been substantiated by degree distributions that follow a highly skewed power-law distribution. Degree refers to the number of nodes to which a given node is immediately connected. Complex networks have no characteristic scales for degree; hence they are called scale-free networks. In such networks, only a few nodes have a very high number of connections and lots of nodes are connected to a few nodes. This phenomenon was first described by Barabási and Albert [25] who have shown that the Web has a scale-free nature. In their experiment more than 80% of the webpages had fewer than four links, but a small fraction of webpages had more than 1,000. Scale-free networks are a class of power-law networks where the high-degree nodes tend to be connected to other high-degree nodes. The power-law fit implies that: (i) the network has no ‘typical’ node, in the sense that a Gaussian distribution would have a mean node; (ii) the distribution is scale-invariant. Many real-world networks have been described with this model, including protein networks, social interactions, and epidemic networks.

Exploiting methods and tools from modern network analysis is part of our long-standing research interest in literature-based discovery [26], [27]. In the present paper, we are concerned with the analysis of biomedical concept co-occurrence structure in the framework of a complex network. In particular, we study co-occurrence associations based on the Medical Subject Headings (MeSH) [28] terminology. MeSH is the controlled vocabulary utilized for indexing, cataloging, and searching articles in the MEDLINE database. The MeSH vocabulary plays an important role in the integration of large-scale biomedical resources. However, the statistical properties of such language structure have not been well studied, particularly in the setting of a complex network. The aim of this study is to fill this gap by investigating the topological properties of a MeSH co-occurrence network in order to understand its global structure. In addition, the network which we are interested in is large-scale and relation datasets of this size have not yet been analyzed in the domain of biomedical research.

## Methods

In the following section, we first introduce some basic terminology of complex networks that we will use, provide information about the process of data collection, and briefly present the techniques we exploit for network analysis.

### Basic Terminology

A network is represented by a graph *G*(*V*, *E*) that consists of a set of nodes *V* representing concepts and a set of edges *E* representing relationships between the nodes. The density of a network is defined as a ratio of the number of edges to the number of all possible edges. We can assign different weights *w_uv_* to the edges, reflecting strength of association between nodes *u* and *v*. The number of edges of a node *i* is denoted by its degree *k_i_*. A path is a sequence of edges which connect a sequence of nodes. For a comprehensive overview of the field of complex networks, see reviews by Newman [18], Bales [14], or Boccaletti et al. [19].

### Data Collection and Network Construction

MEDLINE [1] is the main and largest literature database in the biomedical domain. As of this writing, it contains about 23 million citations dating back to the late 19th century. Since the mid-1940's, MEDLINE citations have been manually annotated using the MeSH vocabulary [28] by trained indexers from National Library of Medicine. MeSH is a controlled vocabulary thesaurus consisting of medical terms at various levels of specificity. There are three types of MeSH terms: main headings (descriptors), supplementary concepts, and qualifiers. Descriptors are the main elements of the vocabulary and indicate the main contents of the citation. For example, for a citation which reports the results of gene expression profiling in the brains of patients who have depressive disorder, MeSH descriptors might be ‘Brain’, ‘Depressive Disorder’, ‘Gene Expression Profiling’, and ‘Humans’. Qualifiers are assigned to descriptors inside the MeSH fields to express a special aspect of the concept. We restrict our analysis to descriptors only. Each MEDLINE citation is manually assigned around 12 MeSH descriptors. In each citation, some MeSH descriptors are designated as major MeSH descriptors. Major descriptors represent the main topic of the citation. The 2013 MeSH, which was utilized in this study, contains 26,853 descriptors.

**Figure 1:**
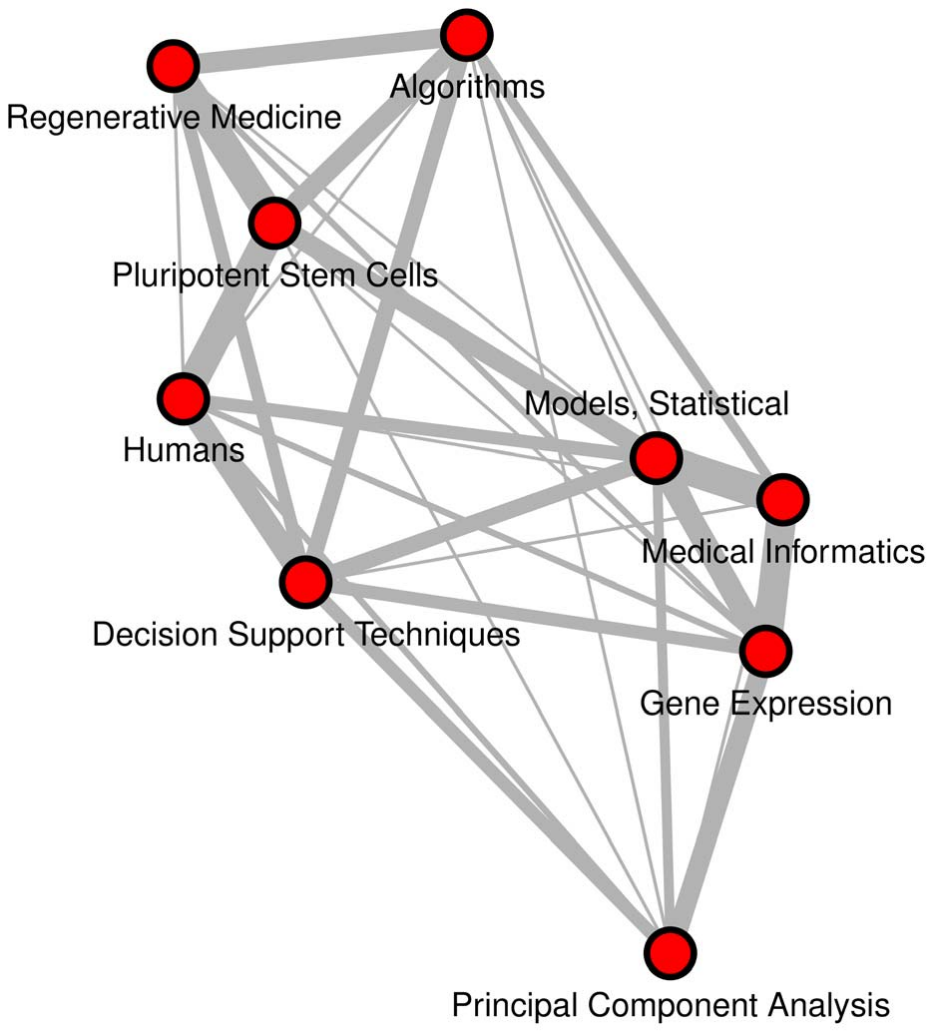
Toy example of the constructed network. Nodes represent MeSH descriptors. An edge between two MeSH descriptors is defined if they appear together in the same MEDLINE citation. Frequency of co-occurrence is represented by edge width. For example, the pair “Medical Informatics” – “Gene Expression” occurs in many more citations then does the pair “Principal Component Analysis” – “Pluripotent Stem Cells”. Note, that we use frequency information only for network reduction purposes (i.e., to obtain a statistic which indicates whether a pair of descriptors occurs together more often than by chance).

We processed the full MEDLINE Baseline Repository, up to the end of 2012, which contains 20,219,186 citations. As the distribution is in XML format, we extracted the relevant elements and transformed them into a relational text format (i.e., one line for each major MeSH descriptor occurrence in each citation). To set up a co-occurrence network, we considered each MeSH term as a node. An edge between two MeSH descriptors was defined if they appear together in the same MEDLINE citation. Note that we did not consider the direction of the relations (i.e., the relation between descriptors *u* and *v* is the same as the relation between descriptors *v* and *u*), that is, the edges are undirected. The network was represented in edge list form. An illustrative example of the constructed network is presented on Figure 1.

The collected network was post-processed to remove all non-useful edges. Descriptors that appear highly frequently (e.g., Humans, Animals, Mice, etc.) and are therefore not useful were removed. We built the list of non-useful MeSH descriptors based on MEDLINE check tags [29]. In addition, we applied the Pearson's chi-square (χ^2^) test for independence [30] for each co-occurrence pair to obtain a statistic, which indicates whether a particular pair of MeSH descriptors occurs together more often than by chance. To the best of our knowledge, this technique is novel in the network analysis community. In the following paragraphs, we provide a detailed description of the chi-square test for independence and its application to network reduction.

For each co-occurrence pair (*u*, *v*) we are interested in co-occurrence frequency and also in the co-occurrences of *u* and *v* with other terms. Complete frequency information is summarized in a contingency table and yields four cell counts (Table 1). *O*_11_ is the joint frequency of the co-occurrence, the number of times the terms *u* and *v* in a co-occurrence are seen together. The cell *O*_12_ is the frequency of pairs in which term *u* occurs, but term *v* does not occur. Likewise, the *O*_21_ is the frequency of pairs in which term *v* occurs, but term *u* does not occur. The cell *O*_22_ is the frequency of pairs in which neither term *u* nor term *v* occurs. The marginal totals are denoted with *R*s and *C*s with subscripts corresponding to the rows and columns. The grand total *N* is the total of all four frequencies (i.e., *O*_11_+*O*_12_+*O*_21_+*O*_22_). Next we calculated the corresponding expected frequencies *E_ij_* for each table cell, as demonstrated in Table 2. Given the observed and expected frequencies for each MeSH descriptor pair, the χ^2^ statistic was calculated as
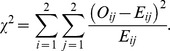



**Table 1:** Contingency table of observed frequencies for pairs of MeSH descriptors.

	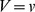	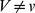	
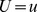	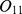	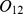	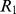
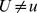	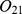	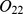	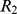
	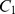	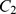	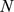

Note: *U* = MeSH descriptor *u*, *V* = MeSH descriptor *v*, *O_ij_* = observed frequency, *R_i_* = row total, *C_j_* = column total, *N* = grand total. For example, cell *O*_12_ refers to the observed frequency of pairs in which descriptor *u* occurs, but descriptor *v* does not occur.

**Table 2:** Calculation of expected frequencies for pairs of MeSH descriptors.

	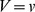	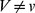
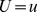	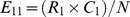	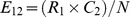
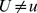	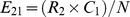	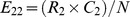

Note: *U* = MeSH descriptor *u*, *V* = MeSH descriptor *v*, *E_ij_* = expected frequency, *R_i_* = row total of observed frequencies, *C_j_* = column total of observed frequencies, *N* = grand total of observed frequencies.

If an expected value was less than five, we applied Yates's correction for continuity by subtracting 0.5 from the difference between each observed frequency and its expected frequency. The limiting distribution of χ^2^ statistic for 2×2 contingency table is a χ^2^ distribution with one degree-of-freedom. If the χ^2^ is greater than the critical value of 3.84 (*p*≤0.05), we can be 95% confident that a particular MeSH relation occurs more often than by chance.

### Network Analysis

We characterize the structure of the MeSH network primarily in terms of four topological features, namely diameter, average path length, clustering coefficient, and degree distribution.

Diameter (*D*) of a network is defined as the maximum distance between all possible pairs of nodes, where distance is the minimum number of edges on the path from one node to another. The diameter is susceptible to outliers. Tauro et al. [31] proposed a more robust measure called integer effective diameter (*D*_90_), which is the minimum number of edges in which at least 90% of all connected pairs of nodes can reach each other. Average path length (*L*) is defined as the mean of the shortest paths between all nodes in a network, namely,
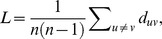
where *n* is the number of nodes in the network and *d_uv_* is the length of the shortest path between nodes *u* and *v*. For most real networks, the average path length is seen to scale with the natural logarithm of the number of vertices in the graph. In addition, compared to random networks the average path length remains small, even if the networks become very large.

The local clustering coefficient (*C_i_*) express the connectedness of the node's neighbors with each other. More formally, *C_i_* is the ratio of the number of edges between its neighbors to the maximal possible number of such edges
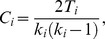
where *T_i_* denotes the number of edges between the neighbors of node *i*, and 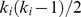 is the number of edges that would be expected between *i*'s neighbors if they formed a fully connected subgraph. The average clustering coefficient *C* is the average of *C_i_* over all nodes in the network, yielding an indicator of the strength of connectivity within the network. The average clustering coefficient captures the global density of interconnected nodes in a network. *C* is normalized to lie in the interval [0, 1]. When *C* = 0, no nodes have neighbors that are also each other's neighbors. In a fully connected network (i.e., every node is connected to all other nodes), *C* = 1. The value of *C* is typically small for random networks (i.e., Erdos-Renyi network), while most real networks exhibit a large average *C*, indicating a high level of connectivity within the network.

Let *L_g_* be the average shortest path length of real network *G* and *C_g_* its clustering coefficient, and let *L_r_* and *C_r_* be the equivalent quantities for the corresponding random network. *G* is said to be small-world network if *L_g_*≈*L _r_* and *C_g_*≫*C_r_*. To express the small-worldness of a network in one parameter, we use a small-worldness index, first introduced by Humphries et al. [32], defined as
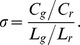



By definition, a small-world network has similar path lengths but greater clustering coefficients when compared with random network. Thus, small-world index is σ>1 if the network has the small-world property.

The degree of a specific node is a local topological feature, and we summarize this information into a global measure of the network by describing the degrees of all nodes in the network in terms of degree distribution. Spread of node degree over a network is characterized by a distribution function *P*(*k*), which is the probability that a randomly chosen node in a network has degree *k*, formally
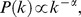
where α is a scaling parameter. The probability of having *k* neighbors is inversely proportional to *k* ^α^. A network that exhibits power-law degree distribution is called a scale-free network. The name ‘scale-free’ comes from the fact that there is no characteristic value of *k*. Such a power law indicates that, while most nodes are sparsely connected, some are linked to many others. Networks are scale-free if the power law holds with an exponent 2<α≤3.

In order to detect power-law behavior we used the rigorous procedure proposed by Clauset et al. [33]. We briefly summarize the algorithm in the next few paragraphs. In practice, the power-law regime applies only for values greater than some minimum lower bound value *k*_min_. We say that the tail of the distribution follows a power law. In this context, it is important to try to find where to start fitting the power-law distribution. First we estimate the parameters α and *k*_min_ of the power-law model. The maximum likelihood estimator of the power-law exponent α is
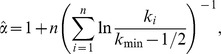
where *k_i_*, *i* = 1, …, *n* are independent observations such that *k_i_*≥*k*_min_. To find the best possible *k*_min_ value, we run through all values and use each degree value as possible *k*_min_ value. We truncate all data below selected *k*_min_. In each iteration, we compute empirical and theoretical cumulative distribution function (CDF). Next we compute the Kolmogorov-Smirnov (KS) statistic by computing the maximum absolute difference between the empirical and theoretical CDF. The KS statistic is calculated as
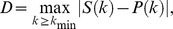
where *S*(*k*) is the CDF of the data for the observations with a value at least *k*_min_, and *P*(*k*) is the CDF for the best fitted model in the region *k_i_*≥*k*_min_. The estimate *k*_min_ is then chosen as a value of *k_i_* for which the KS statistic is the smallest.

Next, we sample a large number of power-law distributed synthetic datasets that follow the power-law model with previously derived scaling parameter α and lower bound *k*_min_. In our computations, we use 2,500 generated datasets as suggested by Clauset et al. [33]. We fit each synthetic dataset to its own power-law model and calculate the KS statistic as described in the preceding paragraph. The fraction of datasets for which the KS statistic is larger than the KS value for the empirical data represents the *p*-value. If the resulting *p*-value is greater than 0.1 the power law is a plausible hypothesis for the data, otherwise it is rejected.

Besides small-worldness and degree distribution, some other measures can help in unraveling in a more detailed manner the topology of the network. We briefly describe assortativity, modularity, and robustness.

The assortativity coefficient of a network measures the probability that nodes link to other nodes of similar degree [18]. The coefficient is calculated as the correlation of the degree of node pairs for all edges in a network and ranges between −1 and 1. A high assortativity coefficient mean that nodes tend to connect to nodes of similar degree, while a negative value means that nodes likely connect to nodes with a very different degree from their own.

Complex networks often exhibit hierarchical structure or modularity, which is characterized by clusters of nodes that are connected to each other through a few long range links. Hierarchical organization of the networks was examined visually by plotting the mean clustering coefficient 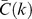 per degree *k*, as described by Ravasz and Barabási [22].

There has been much research interest in the resilience of networks to intentional attacks [34]. Many real-world networks are vulnerable to targeted, but robust to random, attacks. We try to simulate the destructive effect of targeted attack by removing the 10% of nodes with the highest degree and observe the deformation of the giant component. Likewise we performed random attack by random removal of 10% of the nodes.

### Software

Data processing was done using custom Bash and Python scripts. The main part of the network analysis was performed in the R programming language for statistical computing and graphics [35] using igraph and poweRlaw packages. Effective diameters were computed using the SNAP library [36] in C++. The raw data and complete source code to reproduce the results of the analysis is freely available at https://github.com/akastrin/kastrin2014large.

## Results

In this section, we characterize the statistical properties of the MeSH networks. Our experimentation was conducted on two types of co-occurrence networks: (i) the full network, which consists of all MeSH descriptors in each MEDLINE citation and (ii) on the reduced network, which contains only major MeSH terms. First we summarize basic descriptive statistics of the networks and then provide derived topological features.

The full MeSH network consists of |*V*| = 26,385 nodes and |*E*| = 36,597,350 undirected edges. After removal of check tags the network reduced to |*V*| = 26,338 nodes and |*E*| = 36,018,814 edges. The global edge density of the network was ρ = 0.01. We filter out all edges with χ^2^ statistics lower than 3.84. After filtering non-useful relations, the number of edges decreased to |*E*| = 19,408,276. The edge density of the reduced network increased to ρ = 0.06. The largest connected component (i.e., giant component) in which any MeSH term can be reached from any other descriptor contains all nodes of the network. Mean degree of nodes in the giant component was *c* = 1473.79.

The major MeSH network consists of |*V*| = 23,087 nodes and |*E*| = 3,292,926 edges. After removal of check tags the network reduced to |*V*| = 23,039 nodes and |*E*| = 3,226,761 edges. The global density of the network was ρ = 0.01. After filtering redundant relations using χ^2^ test, the number of edges reduced to |*E*| = 2,097,881. The density of the reduced network was ρ<0.01. A giant component comprises |*V*| = 23,023 nodes with |*E*| = 2,097,873 edges. Density of the giant component was ρ<0.01 with a mean degree of *c* = 182.24 nodes. We restricted all further analysis to the giant component.

To establish the small-world property, we first examined the average shortest path length, (effective) diameter and clustering coefficient of both MeSH networks. The average path length between all pairs of nodes in the full network was *L* = 1.95 with a diameter of *D* = 3 edges and 90-percentile effective diameter of *D*_90_ = 1.90 edges. This network exhibits relatively short average path length relative to the number of nodes in the network. That means that, on average, there are only about two hops from the selected node to any other term in the network. The clustering coefficient for exploiting network was *C* = 0.26.

To provide a benchmark for small-world analysis, we also computed the average shortest-path length and clustering coefficient for a random network with size equal to the observed network. Keeping the number of nodes and edges fixed, we compared the results with random graphs generated according to the Erdos-Renyi model. The average path length and clustering coefficient for the random network was *L_r_* = 1.94 and *C_r_* = 0.06, respectively. We observed that the average path length is of the same order as the corresponding random network and the clustering coefficient is seen to be lower than in the original network. The clustering coefficient is about 4.71 times greater than the clustering coefficient of a random network with the same number of nodes and edges. The network has a small-world index of *SWI* = 4.68. With small average path length and high degree of clustering, we conclude that the full MeSH network has the small-world property.

The pattern of statistics is similar for the major MeSH network. The average path length was *L* = 2.63 edges with the diameter of *D* = 7 edges and 90-percentile effective diameter of *D*_90_ = 2.90 edges. The clustering coefficient was *C* = 0.15. The average path length and clustering coefficient for the appropriate random network was *L_r_* = 2.23 and *C_r_* = 0.01, respectively. The network has a small-world index of *SWI* = 16.36, which is considerably higher than in the full MeSH network.

**Figure 2:**
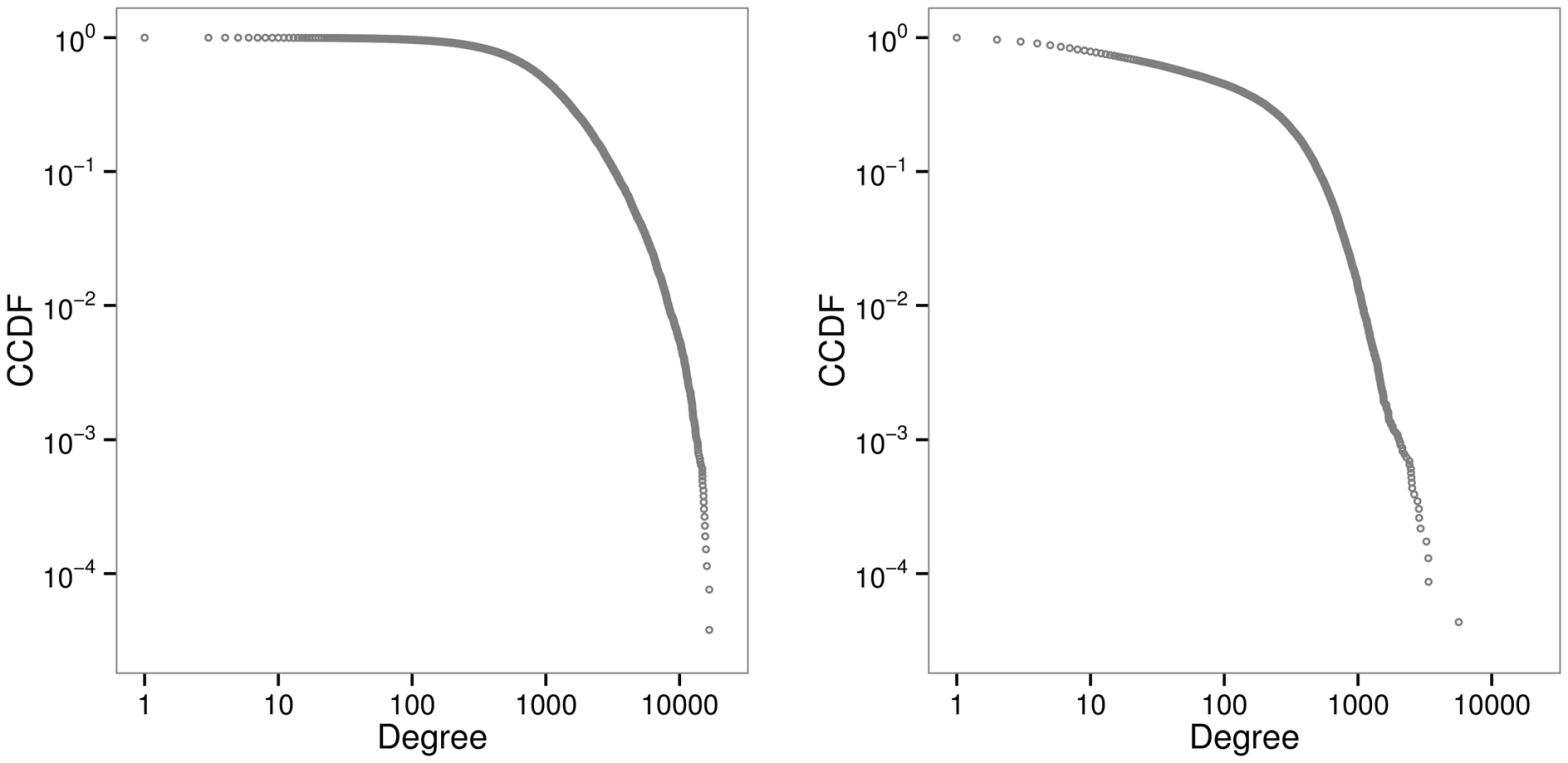
Complementary cumulative degree distribution. The plot shows degree distribution for full (left figure) and major (right figure) MeSH networks.

Next, we examined the degree distribution of the nodes. Figure 2 plots the complementary cumulative degree distribution of the nodes of the network in log-log coordinates. It is evident from the plot that the distribution decays slowly for smaller degree values, while it decays faster for larger degrees.

**Figure 3:**
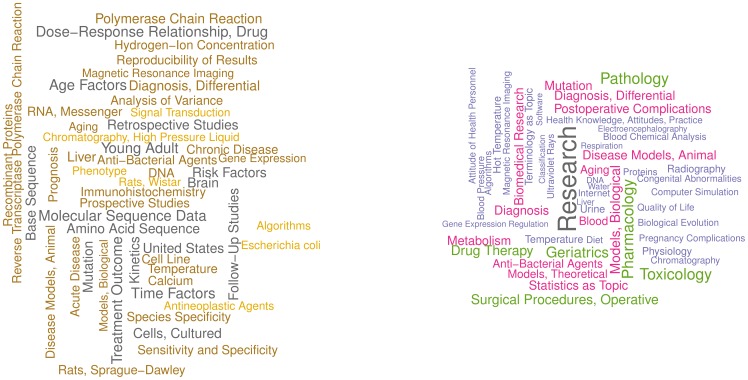
Wordcloud. Visual summary with 50 top degree MeSH descriptors for full (left figure) and major (right figure) MeSH networks. The text size is proportional to the node degree.

It is evident from Figure 2 that the majority of the nodes have a small degree, and a few nodes have a significantly higher degree. For example, in the full MeSH network there are 142 nodes that have degree greater than 10,000 and in the major MeSH network there are 313 nodes with degree greater than 1,000. The high-connectivity terms at the tail of the distributions can be considered as the hubs of the network. For example, the five terms with the highest degree in the major MeSH network are ‘Research’, ‘Child’, ‘Pharmacology’, ‘Pathology’, and ‘Toxicology’. Word clouds with the 50 top degree MeSH terms are depicted in Figure 3.

Besides visual inspection for power law of the degree distribution on Figure 2, we also performed the comprehensive statistical procedure described previously in the Method section. For the full MeSH network the KS test rejects the power law as a plausible model (*D* = 0.05, *p* = 0.002). We conclude that the power-law cannot account for node degree distribution in the full MeSH network. We also cannot adequately fit log-normal (*D* = 0.98, *p*<0.001), Poisson (*D* = 0.65, *p*<0.001) and exponential distribution (*D* = 0.05, *p*<0.001). In the case of the major MeSH network, the KS test failed to reject the power-law model as a plausible model (*D* = 0.02, *p* = 0.603). The exponent α for the best fitting power law was α = 5.07 for nodes with cut-off degree *k*_min_≥941. However, the linear region in the degree distribution spans a limited range of values, which is also evident from the right panel of Figure 2; there were only 410 observations above *k*_min_ degree. Therefore, we cannot conclude that power law is the most plausible model for degree distribution over the entire range of *k* values, although we can state that it provides the best model for the data behavior over a considerable portion of *k* values. We also achieve a very poor fit to the data with log-normal (*D* = 0.57, *p*<0.001), Poisson (*D* = 0.62, *p*<0.001) and exponential distribution (*D* = 0.22, *p*<0.001).

Assortativity was *r* = −0.11 (*p*<0.001) and *r* = −0.04 (*p*<0.001) for the full and major MeSH networks, respectively. Regarding negligible assortativity, we conclude that assortative mixing was not detected in any of the networks considered.

**Figure 4:**
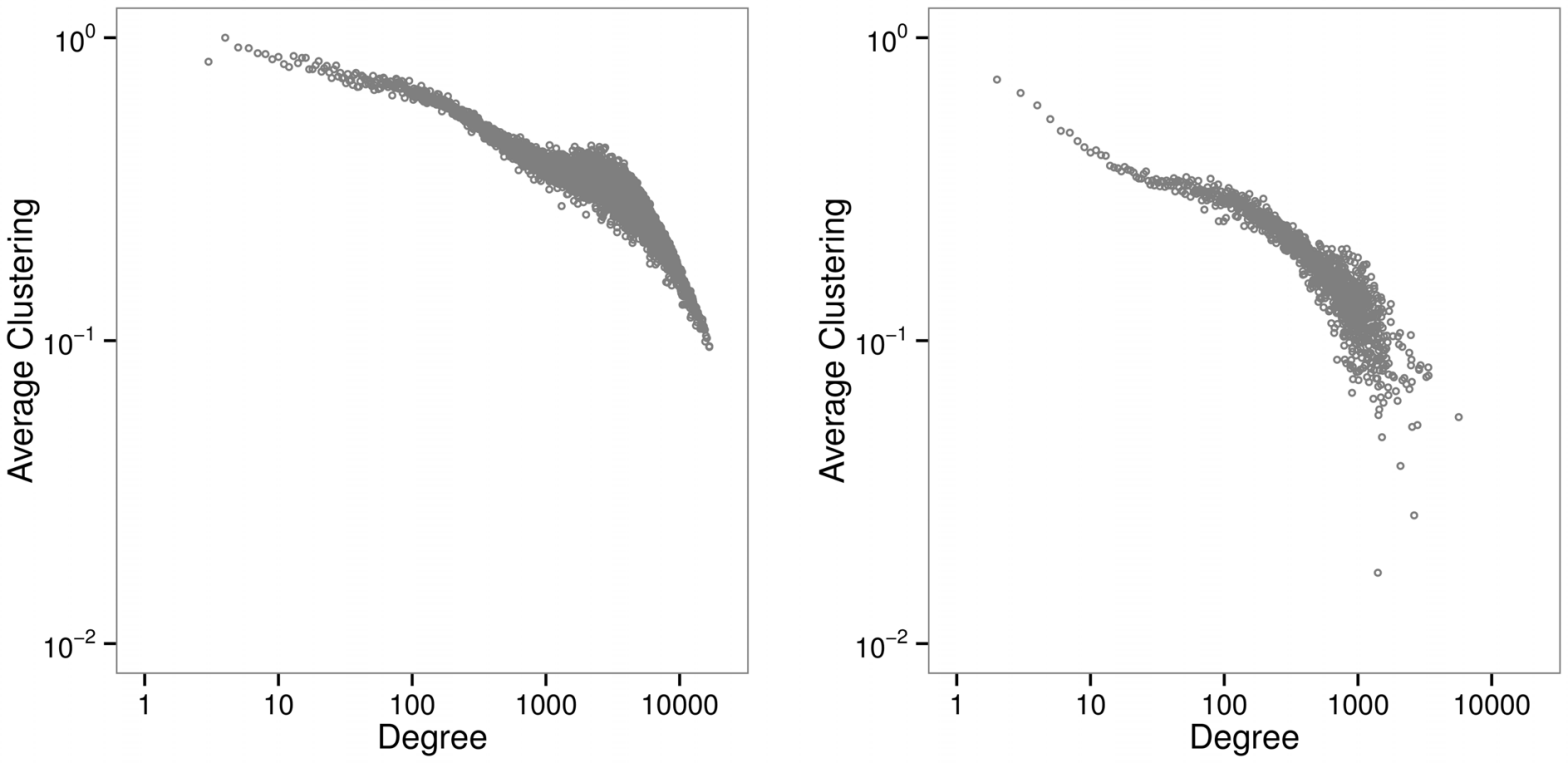
Average clustering per degree. Plot shows average clustering coefficient of nodes per degree for full (left figure) and major (right figure) MeSH networks. The nodes with a smaller degree exhibit higher clustering than those with larger degree. The decay can be approximated by power-law dependency.

In Figure 4, we plot the mean clustering coefficient 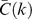 per degree *k* to test for hierarchical architecture of the networks. It is evident that nodes with a lower degree exhibit higher clustering than those with a higher degree. The decay can be approximated by power-law dependency 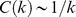, as suggested by Ravasz and Barabási [22]. The nodes with lower degree are essential cornerstones of smaller, densely interconnected clusters, whereas the nodes with higher degree serves as integration units which link together the plethora of smaller clusters into a single network [37].

In most real complex networks, a very large proportion of nodes are connected to each other into a giant component. We showed at the beginning of this section that a giant component includes nearly all nodes of the networks. In network analysis, it is interesting to consider what happens to a giant component when we remove some fraction of nodes. After simulated attack, where we remove 10% of the nodes with the highest degree centrality, the giant component of the full MeSH network contains 90% of the nodes, while for major MeSH network the proportion of nodes decreased to 87% of the initial set of nodes. The pattern of attack tolerance is similar when we remove nodes randomly; the giant component comprises 90% and 89% of the nodes for the full and major MeSH network, respectively. Results are comparable when we based attacks on betweenness centrality. Despite harmful disruption, where we remove the most highly connected nodes from both networks, the networks exhibit a high degree of robustness.

## Discussion

In this paper, we characterize the topological properties of networks based on co-occurrence patterns between MeSH descriptors. We conducted the analysis on networks constructed from both full and major MeSH descriptors. The main findings yielded by the network analysis can be summarized as follows: (i) because of small average path length and high degree of clustering the MeSH network is small-world, (ii) power-law distribution is not a plausible model for the degree distribution, (iii) the network is highly modular, highly resistant to both targeted and random attack and with negligible dissortativity. To the best of our knowledge, this is the first work that investigates the general macroscopic features of a large-scale literature-derived co-occurrence network in the domain of biomedical research.

In general, our findings parallel results obtained in similar studies, particularly in the field of linguistics. Regardless of the large-scale of our networks there exists a relatively short path connecting any pair of nodes within the network. In our case, reaching whatever node involves about three hops on average. For instance, in the major MeSH network we can reach any other term starting from the descriptor ‘Depression’ within three steps on average. In fact, a small-world network does not imply a network where nodes are reciprocal neighbors of each other, but are reached from each other by a small number of hops. We expect the network to be highly clustered, because there are many groups of related terms that are tightly interconnected. For example, the term ‘Microarray Analysis’ is connected to ‘Transcriptome’, and is also connected to ‘Gene Expression Profiling’, and ‘Transcriptome’ and ‘Gene Expression Profiling’ are connected. The small-world nature of the MeSH network can be attributed to the rich relational structure, expressiveness, and universality of the MeSH vocabulary. In the words of cognitive science and linguistics in particular small-worldness also enables fast navigation through the mental lexicon of the MeSH vocabulary [38]. The small-world property may be necessary for an indexer to quickly find an appropriate descriptor among a large number of items in the vocabulary. Similarly, highly clustered nodes could also simplify curation of the MeSH vocabulary. Because related terms are already arranged into interconnected clusters, it should be much easier to identify where new descriptor should be added [39].

The most interesting finding of our study was the poor fit of the power-law model to the degree distributions, which largely contradicts the usual findings in co-occurrence networks [40], [41]. In our case, the power-law function dominates only for a small range of values in the tail region of the distribution for the major MeSH network. We tried to describe the underlying generating process of degree distribution with other common statistical models (i.e., log-normal, Poisson, and exponential), but without a successful fit to the data. Interestingly, similar shape of distribution is found in the famous analysis of the Facebook network of friends, performed by Ugander et al. [42], which is one of the largest networks analyzed to date.

Scale-free behavior emerges from two generic mechanisms: (i) networks grows continuously by addition of new nodes, and (ii) new nodes attach preferentially to nodes that are already well connected [25]. Preferential attachment (PA) means that the more connected the node is, the more likely it is to receive new connections. When the rule of PA is fulfilled, the network exhibits the entire scale-free behavior. Our results clearly suggest that the PA model is too idealized for the MeSH network. An additional argument against attributing the data behavior to the classical PA model is the hierarchical structure of the examined networks, as indicated by the high correlation between node degree and clustering coefficient. It is known that a PA model does not simply yield hierarchical organization [25]. On the contrary, empirical evidence reveals that many real, complex networks have some form of hierarchical structure [22]. This observation is in accordance with rich hierarchical taxonomy of the MeSH terminology. Finding the plausible parametric form (or mixture of them) is a challenge for future work, perhaps indicating novel co-occurrence patterns endemic to MeSH co-occurrence network.

We can envision three limitations of this work. First, the analysis presented relies solely on co-occurrence relations. Co-occurrence represents the simplest way to capture associations between concepts. Co-occurrence, although commonly used, can be interpreted only as an association rather than a substantively meaningful relationship between concepts. Some co-occurrences are also too general to be useful in network modeling. This shortcoming can be overcome by the use of the SemRep system [43] which introduces relevant semantic relationships between concepts (e.g., LRRK2 gene *causes* Parkinson disease). Using SemRep, the relationships between concepts can be described more precisely and with greater semantic expressiveness. Second, our analysis is based solely on static properties, disregarding a temporal view of the network. The MeSH vocabulary is an evolving system, where new terms are constantly created and added to the vocabulary. Dynamic properties (e.g., shrinking diameter, densification power law) should be examined by looking at a series of static snapshots of the network and seeing how statistical indices of these snapshots compare over time. Third, in the network analysis we ignore weights on edges and treat all relationships as equally important.

There are many possible directions for future work. One is to extend the topological analysis on the entire UMLS terminology [44]. Similarly, we are already working on analysis of Semantic MEDLINE [45], a rich network of biomedical concepts and semantic predications between them extracted from titles and abstracts of MEDLINE citations. However, this data is massive and the application of some measures discussed in this paper overwhelms our current computational capabilities. Although these analyses are mainly theoretical, they are unavoidable in the initial stage of data understanding. Finally, the central part of our future research is oriented toward the application of network science in the field of literature-based discovery, where we are interested in discovering meaningful patterns in relational data. To this end, we are currently exploring state-of-the-art machine learning techniques for link prediction in complex networks.

## Conclusions

The aim of this work was to investigate the macroscopic features of a large-scale co-occurrence network based on MeSH descriptors. Analysis was conducted on two networks: (i) a full network consisting of all MeSH descriptors in each MEDLINE citation and (ii) a smaller network containing only major MeSH descriptors. Two MeSH descriptors were connected if they appear together within a MEDLINE citation. We proposed a methodology for reducing the dimensionality of the network, based on the chi-square test for independence. Using the chi-square statistic we obtained a measure which indicates whether a particular pair of MeSH descriptors occurs together more often than by chance. Due to the complexity of graph algorithms (e.g., in huge network the calculation of diameter is infeasible), dimensionality reduction is a crucial preprocessing step. To the best of our knowledge, this approach to network reduction is novel in the network analysis community.

The results of this study demonstrate the small-world nature of the MeSH networks. Both networks have small average path length and high degree of clustering. The power law is not a plausible model for the degree distributions observed. Both networks demonstrated high modularity, which reflects the inherent hierarchical organization of the MeSH vocabulary. Both networks are highly resistant to targeted and random attacks. As far as we know, this is the first analysis of a large-scale literature-derived co-occurrence network in the field of biomedical research. Deeper understanding of network dynamics is the key next step in unraveling the anatomy of the MeSH vocabulary.
